# Population-based assessment of visual impairment among ethnic Dai adults in a rural community in China

**DOI:** 10.1038/srep22590

**Published:** 2016-03-02

**Authors:** Wen-Yan Yang, Jun Li, Chun-Hua Zhao, Deng-Juan Qian, Zhiqiang Niu, Wei Shen, Yuansheng Yuan, Hua Zhong, Chen-Wei Pan

**Affiliations:** 1 Department of Ophthalmology, the First Affiliated Hospital of Kunming Medical University, Kunming, China; 2 Department of Ophthalmology, the Second People’s Hospital of Yunnan Province, Kunming, China; 3 Jiangsu Key Laboratory of Preventive and Translational Medicine for Geriatric Diseases, School of Public Health, Medical College of Soochow University, Suzhou, China

## Abstract

Dai ethnicity is one of the major Chinese ethnic minorities with a population of about 1.2 million. We aimed to determine the prevalence and potential causes of visual impairment (VI) among ethnic Dai adults aged 50 years or older in a rural community in China. A population-based survey including 2163 ethnic Dai people (80.5%) was undertaken using a random cluster sampling strategy. The detailed eye examination was performed after pupil dilation by trained study ophthalmologists and optometrists. Presenting visual acuity (PVA) and best-corrected visual acuity (BCVA) was measured using the Early Treatment Diabetic Retinopathy Study logMAR chart and VI was defined as a VA of less than 20/63 in the better-seeing eye. The overall prevalence of presenting blindness and low vision was 3.0% (95% CI, 2.3–3.7) and 13.3% (95% CI, 11.9–14.8), respectively. The prevalence estimates were reduced to 2.1% (95% CI, 1.5–2.8) and 6.7% (95% CI, 5.7–7.8) when BCVA was considered. Men were more likely to be affected by low vision but less likely to be blind compared with women. Cataract accounted for 62.7% of presenting low vision and 68.8% of presenting blindness, respectively. In conclusion, VI was a significant health concern in Dai Chinese in China.

Visual impairment (VI) is a devastating disability throughout the world^1^,^2^,^3^,^4^,^5^,^6^,^7^ and is associated with functional limitations^8^, falls^9^, difficulties in managing stairs^10^, depressive symptoms^11^, cognitive dysfunction^12^, and increased risk of mortality^13^,^14^. The quality-adjusted life-year (QALY) loss associated with VI has been estimated to be more than other common chronic disorders such as hypertension, diabetes, obesity, and hyperlipidemia^15^. The “Vision 2020: The Right to Sight”, a global initiative proposed by the World Health Organization, has contributed to a reduction of 15 million cases of blindness globally since 1999^16^. However, quite a few people with VI remain undiagnosed and untreated. Recently, the World Health Organization’s report on ‘Universal Eye Health: A global action plan 2014–2019’ highlights the pressing need for regional surveys to obtain data on the burden and causes of VI. It also recommends that the member states target a 25% reduction in the prevalence of VI from 2010 baseline. This underscores the importance of periodic regional surveys to understand both the burdens and the causes in VI over time and to plan strategies to address this issue.

Understanding the epidemiology of VI in the mainland of China may have considerable public health implications as China is the world’s most populous country. The Vision Loss Expert Group of the Global Burden of Disease Study had estimated that the prevalence of VI in China is about 10% in adults aged 50 years or older^1^. However, how accurate the estimation is remains unclear as the calculation was based on several population-based studies in people of Han ethnicity. China has a multiethnic population including Han ethnicity and other 55 ethnic minorities. Han is the major ethnic group which accounts for about 90% of the entire population in China. Although genetic difference may be relatively smaller among different Chinese ethnic groups compared to that among ethnic groups as defined in the United States, the United Kingdom, Australia, and Singapore, there are dramatic variations in cultures, socioeconomic statuses and environmental exposures, which may have a major impact on the pattern and burden of VI among different Chinese ethnic groups. Thus, the findings in Han ethnicity cannot be directly extrapolated to ethnic minorities.

Dai ethnicity is one of the major Chinese ethnic minorities with a population of about 1.2 million (3% of the population in Yunnan), primarily living in Xishuangbanna and Dehong Autonomous Prefectures in Yunnan Province located in the southwestern part of China. All the Dai people believe in Theravada Buddhism. Understanding the epidemiology of VI in Dai people could contribute to the accurate estimation of the disease burden and guide clinical management and health resource allocation in China. In this study, we reported the population-based prevalence and possible causes of VI among middle-aged to elderly adults aged 50 years or older in a rural community in China.

## Methods

### Study cohort and sampling frame

The Yunnan Minority Eye Studies (YMES) are a series of population-based studies aiming at estimating the burden and impact of VI and major age-related eye diseases in ethnic minorities in Yunnan province. In a previous report, we have described the epidemiology of VI in ethnic Bai adults living in Dali city^17^. The study protocol for data collection in Dai ethnicity was the same with that of Bai ethnicity in order to facilitate inter-ethnic comparisons between the two groups. In brief, the study was conducted in Xishuangbanna Autonomous Prefecture in Yunnan province and a random cluster sampling strategy was adopted to select ethnic Dai people aged 50 years or older living in the prefecture. Information regarding ethnicity was collected from the study participants’ identity cards. Each village in the prefecture with a population of approximately 1000 was treated as a sampling cluster. Villages with a population of less than 750 were combined and those of more than 1500 were divided and regrouped. Subsequently, 10% of the total sampling clusters were randomly selected and all the adults of Dai ethnicity aged 50 years or older living in the selected clusters were invited to participate in a detailed eye examination and a face-to-face questionnaire interview. The sampling frame was composed of 111 clusters including 26,328 adults aged 50 years or older, 11 clusters (2671 adults aged 50 years or older) were randomly selected. In the end, 2150 ethnic Dai adults participated in this study with a response rate of 80.5%.

This YMES were approved by the Kunming Medical University Institutional Review Board and the conduct of the studies adhered to the Declaration of Helsinki. The study methods were carried out in accordance with the approved guidelines. Written informed consent was obtained from participants at the recruitment stage.

### Visual acuity (VA) measurement

The protocol for VA measurement has been described in our previous report^17^. In brief, VA was measured in each eye separately by trained study optometrists using the Early Treatment Diabetic Retinopathy Study (ETDRS) logMAR chart with tumbling-E optotypes (Precision Vision, Villa Park, IL) at a distance of 4 m. If the study subject could identify at least 4 out of the 5 optotypes, he or she was then examined by dropping down to line 4 (20/100) line, line 7 (20/50), line 10 (20/25), and finally line 11 (20/20). If the individual failed to identify the top line at 4 m, the subject was advanced to 2 m and then to 1 m, consecutively. If no number could be read at all, VA was assessed as counting fingers, hand movements, perception of light, or no perception of light. The presenting visual acuity (PVA) was ascertained with the participants wearing his or her habitual optical correction, if any. Best-corrected visual acuity (BCVA) was determined after correcting any refractive errors.

### Definitions of VI

Both the World Health Organization (WHO) and the United States (US) definitions were adopted to determine the prevalence of VI in this population. To facilitate inter-ethnic comparison, we followed the study protocol of our previous study on Bai ethnicity to present data^17^. In the WHO definition, VI was defined as a VA of less than 20/63 in the better-seeing eye. Low vision and blindness were defined as a VA <20/63–20/400 and <20/400 in the better-seeing eye, respectively. In the US definition, legal blindness was defined as a VA of not more than 20/200 and low vision was defined as a VA of less than 20/40 but more than 20/200 in the better-seeing eye.

### Eye examinations

The clinical examination procedures have been described previously^17^. Non-cycloplegic autorefraction was performed using an autorefractor (RM-8000; Topcon Corp., Tokyo, Japan). Refraction was subjectively refined until the BCVA was obtained. Intraocular pressure (IOP) was measured using a handheld tonometer (Tono-Pen AVIA; Reichert Inc., Depew, NY) after instilling topical anesthesia (0.4% Benoxil [oxybuprocaine]; Santen Pharmaceuticals, Osaka, Japan). Goldmann applanation tonometry was performed for all glaucoma suspects. The detailed examination of the eyelid, globe, pupillary reflex, lens, and fundi was performed using a slitlamp (model SL-1E; Topcon), a +90-diopter (D) lens at 16 magnification and direct ophthalmoscopy. Individuals whose BCVA of less than 20/40 or whose lens and fundus status could not be examined satisfactorily had their pupils dilated for further examination. Eyes with a PVA of less than 20/40 were assigned a principal cause of VI. Refractive error was considered as the cause of VI for those with a VA ≤20/40 that could be subsequently improved to >20/40 after refractive correction. Cataract was regarded as the main cause of VI if there was no evidence of retinal abnormality in an eye with significant cataract that obscured the vision. Myopic maculopathy was considered only in those with a refractive error exceeding −6.0 D in either eye, in conjunction with one or more of the following ophthalmologic findings: tessellated fundus with yellowish white diffuse or grayish white patchy chorioretinal atrophy, macular hemorrhage, or posterior staphyloma^18^. Age-related macular degeneration (AMD) as a reason for VI was characterized by soft drusen of the retinal pigment epithelium, subfoveal hemorrhage, subretinal and intraretinal edema without any retinal reason detected for it, or a subfoveal disciform scar. Study participants with any of the following signs were identified as glaucoma suspects: the vertical cup-to-disc ratio (VCDR) ≥0.7 in either eye, VCDR asymmetry ≥0.2, a neuroretinal rim width reduced to <0.1 cup-to-disc ratio (between 11 and 1 o’clock or 5 and 7 o’clock), IOP >21 mm Hg, optic disc hemorrhage, notch or nerve fiber layer defect. Glaucoma was defined according to the International Society for Geographical and Epidemiological Ophthalmology Classification^19^. Diabetes was defined as self-reported of a previous diagnosis of the disease, use of diabetic medication, or non-fasting glucose levels greater than 200 mg/dL (11.1 mmol/L). A participant was considered to have type-1 diabetes if the participant was younger than 30 years when diagnosed with diabetes and was receiving insulin therapy. Otherwise, the participant was considered to have type-2 diabetes. Diabetic retinopathy was considered in diabetes patients if the macula showed cystoid macular edema, hard exudates, intraretinal hemorrhages, and microaneurysms.

### Attribution of causes of VI

Primary causes of VI were determined by the trained study ophthalmologists according to clinical examinations, disease definition, and clinical judgment. If cataract and a posterior segment lesion of the optic nerve or retina coexisted and removal of cataract would not restore vision, the cause of VI was considered to be the posterior-segment lesion. If cataract, corneal opacity, or other eye disorders prevented any view of the posterior segment while there was no evidence of any other cause of visual loss, the cause of VI was considered to be cataract, corneal opacity, or other eye disorders. When two or more disorders might have caused VI in same eye, the group of examiners was asked to decide which disease was the main cause. If the two eyes of a subject were visually impaired due to two different causes, the major cause that impaired the vision in the better-seeing eye was treated as the cause for that participant. After the completion of the study, all primary causes of VI were confirmed by a senior investigator (HZ) after reviewing all the eye examination records.

### Statistical Analysis

The Statistical Package for the Social Sciences (SPSS) statistical software (V.16.0; SPSS Inc) was used for data analysis. Age- and gender-specific prevalence rates for VI and their corresponding 95% confidence intervals (CIs) were estimated. The associations of VI and demographic factors including age, gender and education were determined by logistic regression models. Statistical significance was set at a p value of less than 0.05.

## Results

Totally, 2688 ethnic Dai people aged 50 years or older were invited to participate and 2163 (80.5%) were successfully examined. Table 1 shows the study participation rate by age, gender and education level. In general, non-responders were more likely to be younger. For example, the participation rate was only 72% in people aged 50 to 59 years while it was 93.7% in those aged 80 years or older. Non-responders also tended to be men (96.7% vs. 62.5%; comparing women vs. men). The main reasons for not participating were being not interested to participate, being too busy, having good vision, or our inability to contact the subjects. In addition, 13 people were unable to complete VA testing successfully as they could not understand the VA tests or could not cooperate well. Therefore, the analysis of this study was based on the data from 2150 individuals with complete VA data. Education level did not have a major impact on study participation rate.

Table 2 demonstrates the overall prevalence of VI based on PVA and BCVA using both WHO and US definitions. Based on the WHO definitions, the overall prevalence of presenting blindness and low vision was 3.0% (95% CI, 2.3–3.7) and 13.3% (95% CI, 11.9–14.8), respectively. These prevalence estimates were decreased to 2.1% (95% CI, 1.5–2.8) and 6.7% (95% CI, 5.7–7.8) when the data of BCVA was used to define VI. Based on the US criterion, the prevalence of presenting blindness and low vision was 4.2% (95% CI, 3.4–5.1) and 13.7% (95% CI, 12.3–15.2). Unilateral blindness (<20/200 in one eye) was found in 9.1% (95% CI, 7.9–10.4) of the subjects based on PVA, and decreased to 8.6% (95% CI, 7.4–9.7) when BCVA was considered.

Table 3 shows the association of presenting and best-corrected low vision and blindness with age, sex and education. The prevalence of low vision and blindness increased significantly with increasing age. Men were more likely to be affected by low vision but less likely to be blind compared with women. Education level was not significantly associated with the presence of low vision or blindness after adjusting for the effect of age and sex.

Table 4 shows the causes of VI based on the data of eyes by VA and by age groups. As expected, cataract was the major cause for VI, which accounted for more than 60% of the total eyes with VI. Uncorrected refractive error was the second major cause for presenting VI. Other main causes for VI included AMD, myopic maculopathy and glaucoma. When stratified by age, cataract remains the major cause for VI in all age groups. When BCVA was considered, uncorrected refractive error was no longer a cause for VI and AMD was the second cause for VI only secondary to cataract.

Table 5 shows the causes of presenting and best-corrected VI based on the data of persons. Cataract accounted for 62.7% of presenting low vision and 68.8% of presenting blindness, respectively. When defined by BCVA, cataract remained the leading cause of VI (68.6%) while AMD and myopic maculopathy accounted for 8.9% and 7.3% of VI in this cohort.

## Discussion

In this population-based eye survey of middle-aged to elderly adults of Dai ethnicity in rural China, we reported that 15.9% of the study participants were affected by presenting VI and 8.0% were affected by best-corrected VI, which was a bit lower compared with the rates of Bai ethnicity living in the same province. The major causes of presenting VI in this population were cataract, uncorrected refractive error and age-related macular degeneration. Our results contribute to the accurate estimation of the burden of VI in mainland of China.

Comparing prevalence estimates among different studies should account for methodological disparities among different studies such as sampling strategies, definitions of the outcomes, and age ranges of the study subjects. This study used the same study protocol for data collection and analysis as we previously used in Bai ethnicity so that the results of the two ethnic groups could be directly compared. The prevalence of presenting VI was lower compared with that in our previous report of Bai ethnic group (19%)^17^. Cataract surgery is an important contributing factor when estimating the prevalence of VI. We have noticed that the prevalence of cataract surgery in Dai ethnicity was higher than that in Bai ethnicity. Cataract surgery rate was only 4.6% in ethnic Bai people^20^ but was 8.9% in ethnic Dai people. Thus, higher cataract surgery rates might have explained the lower prevalence of VI in Dai compared with Bai adults. In addition to the disparities in cataract surgery rates, ethnicity also reflects other population-wide variations in variables such as religion, diet and culture. Up till now, there have been few data addressing the potential roles of these changes as risk factors for VI, and one can only speculate which of these variables, if any, might be related to the risk of VI.

The Vision Loss Expert Group of the Global Burden of Disease Study had estimated that VI affects about 10% of the people aged 50 years or older in China^1^. The estimates were primarily based on the results of Han ethnicity. The current study together with our previous study on Bai people showed that the prevalence of VI was higher in ethnic minorities than Han Chinese. Chinese government always places a lot of emphasis on “national minorities” and the need for affirmative action to enhance their social situation while maintaining their identity. Thus, the health status of ethnic minorities should not be neglected and health inequities between the major and minor ethnic groups should be properly addressed. Efforts and resources could be channeled towards the prevention and treatment of visually-disabling ocular complications in ethnic minorities. Our results may also have implications for other countries with multiethnic populations where the main objective of public health reform is to readdress the health inequalities among different ethnic groups.

It was not surprising that cataract was the leading cause of VI in ethnic Dai people, which was consistent with other population-based surveys throughout the world^21^,^22^,^23^,^24^,^25^,^26^,^27^,^28^. Our study indicated that approximately 70% of VI cases were explained by the presence of cataract and this proportion increased with age. Our study participants were poorly educated with low socioeconomic status, who had limited knowledge in eye healthcare. Most of them were farmers and fishers, who spent most of their working hours outdoors and were intensively exposed to sunlight. Thus, we think that free cataract surgery service should be provided by the local government on a routine basis to reduce the burden of VI associated with cataract in ethnic minorities residing in Yunnan.

The current study had several strengths including a population-based study sample, reasonable response rate, and the use of standardized protocols for data collection. The results were comparable with other population-based studies. Our study was also one of the few studies which added knowledge on the epidemiology of VI among ethnic minorities in China. Meanwhile, there were still some limitations which should be acknowledged. Responders and non-responders were different in terms of age and gender distribution, which may bias the results. Non-responders were younger so that the prevalence of VI could have been overestimated considering the increasing trend of VI with increasing age. In addition, severe lens and corneal opacity may prevent ophthalmologists from detecting posterior-segment eye diseases such as retinal pathology, myopic maculopathy, AMD, and glaucoma. Thus, the prevalence of VI associated with posterior-segment eye diseases may have been underestimated. Finally, visual fields tests were not performed on all participants because of logistics constraint and constricted fields were not included in the definition of blindness. Therefore, the prevalence of blindness associated with glaucoma and peripheral retinal disease might have been underestimated.

In conclusion, our study suggests that VI including blindness is now a significant public health concern in middle-aged to elderly adults of ethnic minorities in China. Further estimation on the Chinese national burden of VI should include the results from ethnic minorities to get a more accurate estimate and better guide clinical management and health resource allocation. Health policy makers should be aware of these findings and appropriately address the health inequalities in different ethnic groups in China.

## Additional Information

**How to cite this article**: Yang, W.-Y. *et al.* Population-based assessment of visual impairment among ethnic Dai adults in a rural community in China. *Sci. Rep.* **6**, 22590; doi: 10.1038/srep22590 (2016).

## Figures and Tables

**Table 1:** Study Population by Age, Gender and Education.

	Enumerated No. (%)	Examined No. (%)	% Examination Response Rate
Age (years)			
50–59	1167(43.4)	840(38.8)	72.0
60–69	810(30.1)	695(32.1)	85.8
70–79	536(19.9)	464(21.5)	86.6
80 or older	175(6.5)	164(7.6)	93.7
Gender			
Male	1276(47.5)	797(36.8)	62.5
Female	1412(52.5)	1366(63.2)	96.7
Education			
Illiteracy	2047(76.2)	1632(75.5)	79.7
Primary school	601(22.4)	500(23.1)	83.2
Middle school	33(1.2)	26(1.2)	78.8
High school or higher	7(0.3)	5(0.2)	71.4
All	2688(100.0)	2163(100.0)	80.5

**Table 2:** Prevalence of Visual Impairment Based on PVA and BCVA.

Category	VA Definition		Prevalence With PVA		Prevalence With BCVA	
	Better Eye	Worse Eye	No. (%)	[95% CI]	No. (%)	[95% CI]
NN	≥20/63	≥20/63	1523(70.8)		1734(80.7)	
VI	≥20/200	<20/63 to ≥20/200	341(15.9)	(14.32–17.41)	173(8.0)	(6.90–9.20)
UL	≥20/200	<20/200	197(9.12)	(7.94–10.38)	184(8.6)	(7.37–9.74)
MB	<20/200 to ≥20/400	<20/200	25(1.2)	(0.71–1.62)	13(0.6)	(0.28–0.73)
SB^a^	<20/400	<20/400	64(3.0)	(2.26–3.70)	46(2.1)	(1.53–2.75)
WHO low vision	<20/63 to ≥20/400		287(13.3)	(11.91–14.79)	145(6.7)	(5.68–7.81)
US blindness	≤20/200		160(7.4)	(6.33–8.55)	91(4.2)	(3.38–5.08)
US low vision	<20/40 to >20/200		511(23.8)	(21.97–25.57)	295(13.7)	(12.27–15.18)
Total			2150		2150	

Abbreviations: BCVA, best-corrected visual acuity; CI, confidence interval; MB, moderate bilateral blindness; NN, normal/near normal; PVA, presenting visual acuity; SB, severe bilateral blindness; UL, unilateral blindness; VA, visual acuity; VI, vision impairment; WHO, World Health Organization.

^a^Equivalent to WHO blindness definition (VA < 20/400 in better eye).

**Table 3:** Presenting and Best-corrected Visual Impairment by Age, Gender and Education.

	No.	Presenting Low Vision		Presenting Blindness		Best-Corrected Low Vision		Best-Corrected Blindness	
		Prevalence	Adjusted Odds Ratio	Prevalence	Adjusted Odds Ratio	Prevalence	Adjusted Odds Ratio	Prevalence	Adjusted Odds Ratio
		No. (%)	(95% CI)	No. (%)	(95% CI)	No. (%)	(95% CI)	No. (%)	(95% CI)
Age (yrs)									
50–59	840	31(3.69)	—	5(0.60)	—	15(1.79)	—	2(0.24)	—
60–69	695	67(9.64)	2.86(1.83–4.4)^†^	13(1.87)	2.96(1.03–8.46)^*^	26(3.74)	2.31(1.20–4.46)^†^	9(1.29)	4.91(1.03–23.10)^*^
70–79	451	128(28.38)	10.45(6.77–16.12)^†^	22(4.88)	8.04(2.92–22.15)^*^	64(14.19)	9.97(5.42–18.33)^†^	18(3.99)	15.51(3.47–69.40)^*^
≥80	164	61(37.20)	15.28(9.25–25.23)^†^	24(14.63)	27.87(9.99–77.75)^†^	40(24.39)	19.26(9.92–37.41)^†^	17(10.37)	44.80(9.82–204.21)^†^
Gender									
Female	1360	203(14.93)	—	35(2.57)	—	105(7.72)	—	24(1.76)	—
Male	790	84(10.63)	0.70(0.52–0.93)^*^	29(3.67)	1.71(1.01–2.90)^*^	40(5.06)	0.66(0.45–0.99)^*^	22(2.78)	1.92(1.04–3.52)^*^
Education									
Primary or higher	531	37(6.97)	—	7(1.32)	—	20(3.77)	—	4(0.75)	—
Illiterate	1619	250(15.44)	0.99(0.66–1.49)	57(3.52)	1.217(0.52–2.87)	125(7.72)	0.78(0.45–1.34)	42(2.59)	1.45(0.49–4.35)

^*^P < 0.05.

^†^P < 0.0001.

CI = confidence interval.

**Table 4:** Principal Cause of Impairment in Eyes with Presenting and Best-corrected Visual Acuity Less Than 20/63^a^.

Principal Cause	NO. (%)							Total
	Eyes by Visual Acuity			Eyes by Age, y			≥80	
	<20/63 to ≥20/200	<20/200 to ≥20/400	<20/400	50–59	60–69	70–79		
Cataract	383(63.5)	32(50.0)	181(58.2)	35(31.0)	129(51.0)	279(69.1)	153(73.6)	596(61.0)
	203(66.6)	15(45.5)	158(58.7)	16(25.8)	67(49.6)	181(70.2)	112(73.7)	376(61.9)
Refractive error	95(15.8)	1(1.6)	0(0.0)	20(17.7)	43(17.0)	29(7.2)	4(1.927)	96(9.8)
AMD	42(7.0)	3(4.7)	15(4.8)	1(0.9)	9(3.6)	36(8.9)	14(6.7)	60(6.1)
	40(13.1)	0(0.0)	12(4.5)	1(1.6)	7(5.2)	31(12.0)	13(8.6)	52(8.6)
Myopic maculopathy	10(1.7)	11(17.2)	15(4.8)	22(19.5)	10(4.0)	4(1.0)	0(0.0)	36(3.7)
	11(3.6)	7(21.2)	10(3.7)	15(24.2)	9(6.7)	4(1.6)	0(0.0)	28(4.6)
Glaucoma	8(1.3)	2(3.1)	15(4.8)	0(0.0)	4(1.6)	13(3.2)	8(3.8)	25(2.6)
	5(1.6)	0(0.0)	15(5.6)	0(0.0)	4(3.0)	9(3.5)	7(4.6)	20(3.3)
Corneal opacity	3(0.5)	5(7.8)	24(7.7)	12(10.6)	8(3.2)	7(1.7)	5(2.4)	32(3.3)
	4(1.3)	5(15.2)	23(8.6)	12(19.4)	8(5.9)	7(2.7)	5(3.3)	32(5.3)
Pterygium	22(3.6)	2(3.1)	10(3.2)	5(4.4)	19(7.5)	6(1.5)	4(1.9)	34(3.5)
	9(3.0)	1(3.0)	10(3.7)	1(1.6)	12(8.9)	4(1.6)	3(2.0)	20(3.3)
Vascular retinopathy	5(0.8)	3(4.7)	8(2.6)	3(2.7)	9(3.6)	2(0.5)	2(1.0)	17(1.7)
	4(1.3)	2(6.1)	6(2.2)	2(3.2)	8(5.9)	2(0.8)	0(0.0)	12(2.0)
Undetermined	3(0.5)	2(3.1)	3(1.0)	6(5.3)	0(0.0)	0(0.0)	2(1.0)	8(0.8)
	5(1.6)	0(0.0)	3(1.1)	6(9.7)	0(0.0)	0(0.0)	2(1.3)	8(1.3)
Others	32(5.3)	3(4.7)	40(12.9)	9(8.0)	22(8.7)	28(6.9)	16(7.7)	75(7.7)
	24(7.9)	3(9.1)	32(11.9)	9(14.5)	20(14.8)	20(7.8)	10(6.6)	59(9.7)
All	603(100.0)	64(100.0)	311(100.0)	113(100.0)	253(100.0)	404(100.0)	208(100.0)	978(100.0)
	305(100.0)	33(100.0)	269(100.0)	62(100.0)	135(100.0)	258(100.0)	152(100.0)	607(100.0)

^a^For each principal cause, the first row represents the result based on presenting visual acuity while the second row represents the result based on best-corrected visual acuity. AMD = age-related macular degeneration.

**Table 5:** Principal Causes of Low Vision and Blindness in Persons with Presenting and Best-Corrected Visual Impairment.

Causes	Based on PVA						Causes	Based on BCVA					
	Visual							Visual					
	Low Vision#		Blindness*		Impairment‡			Low Vision#		Blindness*		Impairment^‡^	
	(n = 287)		(n = 64)		(n = 351)			(n = 145)		(n = 46)		(n = 191)	
	No.	%	No.	%	No	%		No.	%	No.	%	No.	%
Cataract	180	62.7	44	68.8	224	63.8	Cataract	97	66.9	34	73.9	131	68.6
Refractive error	34	11.8	0	0.0	34	9.7	AMD	16	11.0	1	2.2	17	8.9
AMD	19	6.6	3	4.7	22	6.3	Myopic maculopathy	11	7.6	3	6.5	14	7.3
Myopic maculopathy	13	4.5	6	9.4	19	5.4	Corneal opacity	4	2.8	2	4.3	6	3.1
Glaucoma	6	2.1	4	6.3	10	2.8	Glaucoma	1	0.7	4	8.7	5	2.6
Pterygium	8	2.8	0	0.0	8	2.3	Pterygium	4	2.8	0	0.0	4	2.1
Corneal opacity	3	1.0	3	4.7	6	1.7	Vascular retinopathy	1	0.7	1	2.2	2	1.0
Vascular retinopathy	4	1.4	1	1.6	5	1.4	Others	11	7.6	1	2.2	12	6.3
Others	20	7.0	3	4.7	23	6.6							

Abbreviation: PVA, presenting visual acuity; BCVA, best-corrected visual acuity; AMD, age-related macular degeneration.

^*^Defined as visual acuity <20/400.

^#^Defined as visual acuity <20/63 and ≥20/400.

^‡^Blindness plus low vision, defined as visual acuity <20/63.
